# Unleashing the Power of IoT: A Comprehensive Review of IoT Applications and Future Prospects in Healthcare, Agriculture, Smart Homes, Smart Cities, and Industry 4.0

**DOI:** 10.3390/s23167194

**Published:** 2023-08-16

**Authors:** Robin Chataut, Alex Phoummalayvane, Robert Akl

**Affiliations:** 1 School of Computing and Engineering, Quinnipiac University, Hamden, CT 06518, USA; 2 Computer Science Department, Fitchburg State University, Fitchburg, MA 01420, USA; aphoumma@student.fitchburgstate.edu; 3 Department of Computer Science, University of North University, Denton, TX 76203, USA; robert.akl@unt.edu

**Keywords:** IoT, smart cities, internet-of-medical-things, sensors, security, Industry 4.0

## Abstract

The Internet of Things (IoT) technology and devices represent an exciting field in computer science that is rapidly emerging worldwide. The demand for automation and efficiency has also been a contributing factor to the advancements in this technology. The proliferation of IoT devices coincides with advancements in wireless networking technologies, driven by the enhanced connectivity of the internet. Today, nearly any everyday object can be connected to the network, reflecting the growing demand for automation and efficiency. This paper reviews the emergence of IoT devices, analyzed their common applications, and explored the future prospects in this promising field of computer science. The examined applications encompass healthcare, agriculture, and smart cities. Although IoT technology exhibits similar deployment trends, this paper will explore different fields to discern the subtle nuances that exist among them. To comprehend the future of IoT, it is essential to comprehend the driving forces behind its advancements in various industries. By gaining a better understanding of the emergence of IoT devices, readers will develop insights into the factors that have propelled their growth and the conditions that led to technological advancements. Given the rapid pace at which IoT technology is advancing, this paper provides researchers with a deeper understanding of the factors that have brought us to this point and the ongoing efforts that are actively shaping the future of IoT. By offering a comprehensive analysis of the current landscape and potential future developments, this paper serves as a valuable resource to researchers seeking to contribute to and navigate the ever-evolving IoT ecosystem.

## 1. Introduction

The Internet of Things technology revolves around the core concept of integrating sensors into everyday objects and using connectivity to facilitate the exchange of information that is used in a variety of applications [1]. There are more everyday objects available than people, so the amount of connectivity that IoT devices hold is enormous [2]. In order to better understand the future of IoT technology, it is important to understand the unique circumstances that brought IoT to this point. A key distinction to make between the internet and the IoT is that the internet is a mesh of networks, whereas the IoT network is an interconnected network of devices [3,4]. An early example of the first IoT device was John Romkey’s creation, which enabled a toaster to be turned on or off over the internet in 1990 [5]. It is clear that Internet of Things devices have come a long way from their humble beginnings, and there are many factors that influenced this rise. These devices play an important role in people’s daily lives and involve the handling of massive amounts of data [6]. IoT devices can be seen as a network of interconnected devices that involves sending and actuating devices that provide the ability to share information across different platforms [7,8,9,10,11,12,13,14,15,16,17,18,19,20,21].

**Figure 1:**
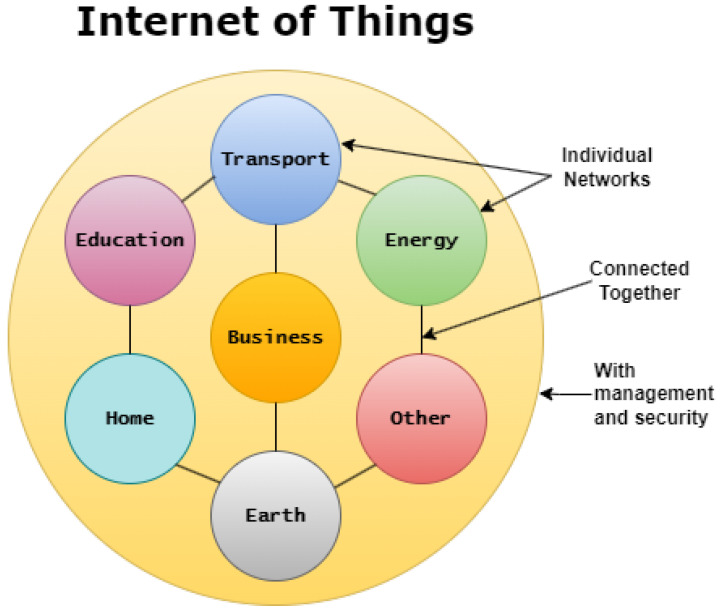
IoT technology and diverse application domains.

The rapid rise in technology and computer system capabilities has had a major impact on the proliferation of Internet of Things (IoT) technology. The impact of IoT systems will impact many different fields and will change the way society operates and moves toward the future, as shown in Figure 1. This paper discusses the use of IoT in four domains. As IoT devices become increasingly integrated into society, there are still numerous security challenges that pose a threat to their spread. Numerous technologies are being developed in order to help protect the safe use of IoT devices, and it is clear that advances in security will be critical moving forward into the future [22,23,24,25,26,27].

Numerous research works have been conducted to address various aspects of the Internet of Things (IoT), encompassing energy harvesting, device-to-device communication, energy efficiency, resource allocation, edge computing, security, privacy, and applications across different domains. Scholars have explored stochastic geometry analysis to optimize energy harvesting, proposed energy-efficient systems, investigated untapped potential in spectrum utilization, developed reinforcement learning-based frameworks, and explored the integration of edge computing and artificial intelligence [28,29,30,31,32,33,34,35,36,37,38,39,40,41,42]. Additionally, studies have focused on addressing security concerns in these areas, including privacy-preserving data sharing, explainable AI, and motion tracking. Collectively, these research efforts contribute to a comprehensive understanding of IoT technologies and their applications in diverse fields [43,44,45,46,47,48,49,50,51].

Many different strategies are being utilized by computing professionals in order to advance the field of research in IoT technology [52,53,54,55,56]. In particular, machine learning and artificial intelligence have played key roles in advancing this emerging field of computer science and will continue to do so in new and exciting ways [57]. It is critical for computer scientists to understand common machine learning and AI algorithms that are being used, as well as consider what research is currently being conducted to advance IoT technologies [58,59,60,61,62,63,64,65,66,67,68,69,70,71,72,73].

The methodology followed for conducting the review involves a systematic literature search, a study selection based on predefined criteria, data studies, a critical evaluation of the selected studies, and a comprehensive discussion of the findings. This review aims to provide an overview of IoT applications and future directions in the selected domains, ensuring the inclusion of relevant research and a rigorous evaluation process.

## 2. Applications of IoT Devices

**Figure 2:**
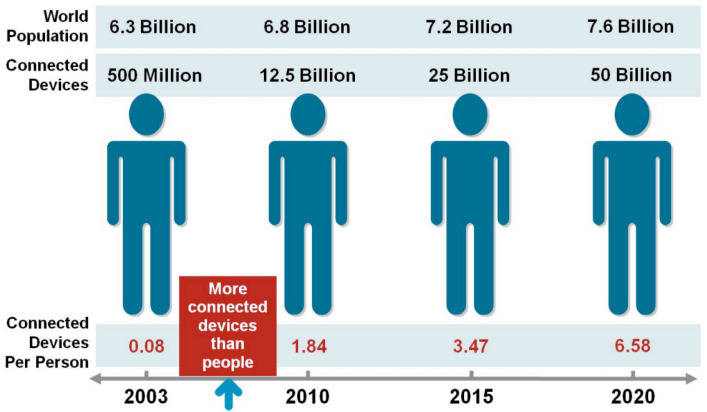
The number of IoT devices is greater than the number of people [82].

**Figure 3:**
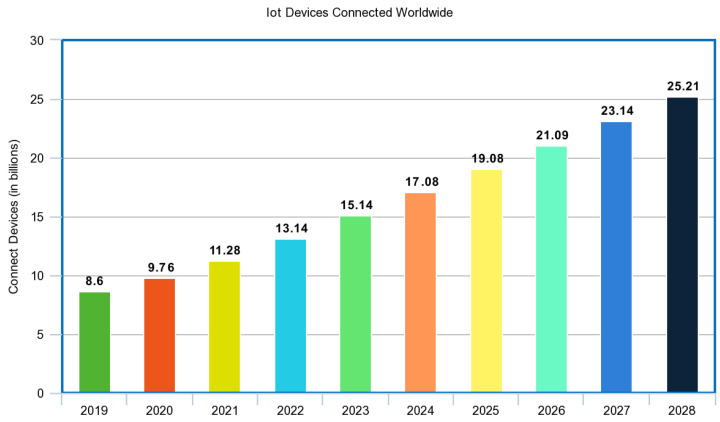
Number of IoT devices globally [83].

**Figure 4:**
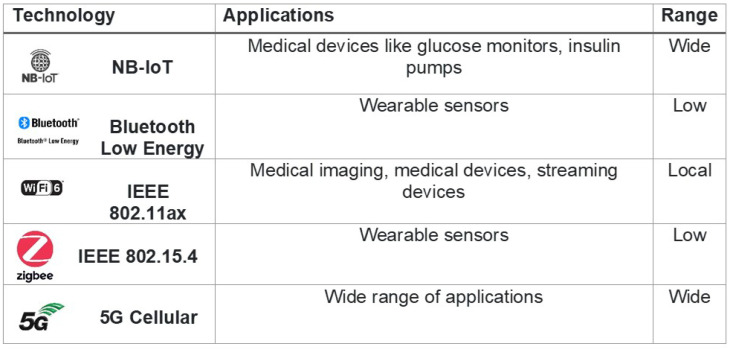
Connectivity in different medical IoT devices.

Since Internet of Things technology can be understood as devices/objects that are connected to a network, the applications of IoT devices are endless [74,75,76,77,78,79,80]. Leaders of different companies/organizations are recognizing the potential of IoT technology to make an impact and are investing more in these key pieces of technology in order to reap the benefits [81]. Nearly any object can be outfitted with the appropriate technology that will be involved in the data transmission from IoT devices and their connected networks. Writing about the different applications would be a long and arduous process and it would be more beneficial to first understand the most common applications of IoT devices before exploring what the future holds. Figure 2 and Figure 3 shows growth in IoT globally. The connectivity in different medical IoT devices is shown in Figure 4.

### 2.1. Healthcare Applications

The Internet of Medical Things (IoTM) is an emerging subfield that is changing the way healthcare is being delivered through the use of IoT technology (Joyia). The use of IoT technology in healthcare has come a long way and continues to be a promising area for growth. Essential innovations, such as the AliveCor heart monitor, which relies on IoT sensors, show how useful technology can be when applied to healthcare in efforts to save lives [84]. Advances in technology have consistently played a major role in the healthcare industry, and IoT devices have found numerous applications in healthcare settings. One way that IoT devices are useful in healthcare is through the use of remote health monitoring in order to monitor patients at home rather than in hospitals [85]. The information that is collected from IoT devices is helpful in medical settings because it can be analyzed and used in ways such as early disease prediction [86]. IoT sensors even played a critical role during the COVID-19 pandemic in helping healthcare workers better monitor critical parameters that could save lives if changes were detected right away [87]. By examining these different IoT device applications in the healthcare industry, researchers can find additional ways to advance this field of research.

The use of sensors is critical in the delivery of healthcare services [88]. Sensors in medical devices act as a bridge between the physical and information worlds by collecting a variety of data. Sensors are crucial in helping healthcare professionals monitor different vitals that are important to measure in order to understand a person’s health situation and act accordingly. Medical sensors can be used in a variety of ways in order to measure crucial information. Medical sensors are connected to IoT, and measure things such as temperature, respiration, heart rate, weight, skin conductance, galvanic response, blood flow/SpO2, glucose testing, muscle contraction, and motion analysis [89]. The medical sensors are connected to wireless sensor networks, which relay useful information to different stakeholders involved in healthcare, such as patients, medical staff, insurers, and more [90]. The use of medical sensors is vast and can be used in crucial medical equipment, such as ECG monitors, glucose level sensing, and oxygen monitoring [91]. The goal of using any technology involved in healthcare is to promote better health outcomes, and IoT devices play a critical role in promoting this. Recent advances in IoT-related technology will continue to play a large role in creating stronger healthcare systems, and the future of healthcare will become increasingly reliant on technology [92].

Medical sensors are important in collecting useful information about a patient’s health; however, this information is often very sensitive in nature, and this makes privacy a major concern moving forward. Security has always played a vital role in IoT technology; however, it matters even more in a situation such as healthcare, where IoT devices will be collecting sensitive information about patients that is private in nature [93]. If a patient’s medical information was compromised, this could lead to consequences for hospital organizations that did not employ the proper security measures to prevent it. The privacy and confidentiality of a patient’s medical information are core concerns when addressing the security vulnerabilities of healthcare IoT devices [94].

There are many issues that present challenges to the successful use of IoT devices in healthcare and it is important to address these issues thoroughly when handling mission-critical operations, such as that of healthcare. Some important limitations that influence the use of IoT technology in medical devices include the need for high power consumption, the availability of limited resources, and handling security issues from the large number of devices being used [95].

The use of IoT technology in healthcare is promising and exciting. There are many useful applications where IoT devices can be used as sensors, and this helps healthcare providers in a variety of ways. Moving forward, IoT technology will continue to expand, and this will ultimately benefit healthcare organizations.

### 2.2. Agriculture Applications

**Figure 5:**
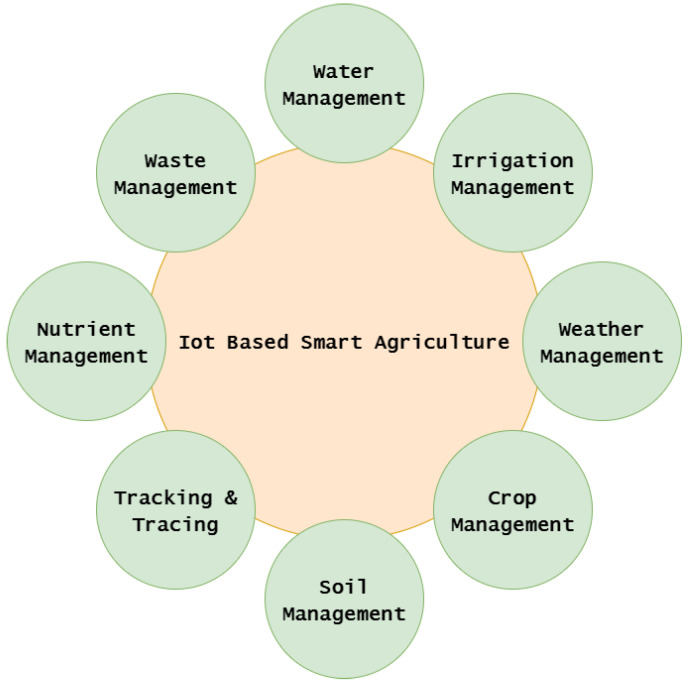
Different types of agriculture applications for IoT.

As the population of the world grows at an exponential rate, the need for efficient food delivery systems is becoming a core issue that is a driver behind the advancements in smart agriculture [96]. In addition to the growing demand, factors such as climate change and water scarcity have also played roles in the increasing demand for more efficient agriculture systems [97]. Much of the technology around IoT implementation aims to reduce agricultural resource waste [98] as shown on Figure 5. The use of IoT technology in agricultural settings is critical to maintaining efficient operations and represents another common use case of IoT technology. Food supply chains that deliver quality and quantity are important to feed the world, and having efficient systems built around these supply chains will benefit people all over the world [99]. The need for more efficient food-delivery systems has helped to promote IoT use in agriculture because stakeholders saw the benefits that technology could provide [100].

One way that IoT technology can be used in agriculture is automation. Automation involves having devices/objects respond automatically to different conditions without the need for human interaction. Wireless sensor networks are key proponents in helping IoT devices achieve their automation goals [101]. This can be useful in massive operations, such as agriculture, because of its sheer scale and need for efficient processes to maximize crop yields. For example, many sensors can be used in the soil of farmland in order to measure soil moisture content, in order to build systems that make better use of water for irrigation purposes [102]. These IoT devices can be used to measure soil conditions, such as water content, give appropriate signals when it is low, and turn on sprinkler systems automatically. Real-time monitoring and responses are very common and useful when understanding how IoT devices contribute to agriculture [103].

Data analytics is another area where IoT plays an important role in agriculture. Collecting and analyzing data are very useful because they can give important insight into how effective or ineffective an operation is. These data can be used to provide stakeholders with important insight that will ultimately impact their decision-making [104]. IoT devices collect massive amounts of data, and these data are useful when analyzed over time to help aid in decisions about estimation and forecasting [105]. The gathered data can be analyzed using machine learning methods that impact prediction, storage management, decision-making, farm management, and precision farming [96]. These data can become useful when attempting to implement more sustainable farming methods through the use of data-driven decision-making [104].

Although there is a large demand for efficient agriculture, there are other factors in play that have affected the proliferation of IoT devices in this sector. One key component that affects how widespread IoT devices are in agricultural applications is how costly it is to implement them in farming operations around the world [106]. Massive farming operations would require a large number of wireless sensors to collect data about a farming operation, and this can drastically increase the costs associated with implementing IoT in agriculture [107]. There are also many technical challenges that exist with implementing IoT technology in farms. Farms are often in large areas that are isolated and usually have poorer signals that impact their networking capabilities [108]. In addition to this, many farmers in rural parts of the world have limited knowledge of how to use IoT devices [109].

### 2.3. Smart Home Applications

**Figure 6:**
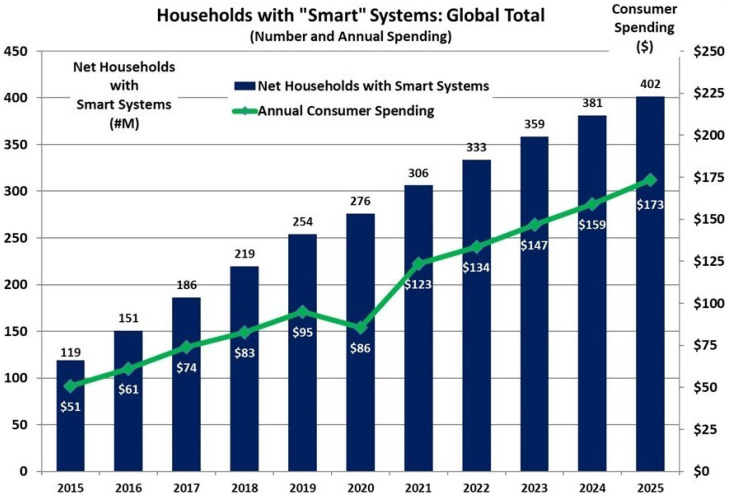
Households with smart systems: global total [115].

Smart home applications represent promising use cases in which people benefit from IoT technology and there are numerous advantages/disadvantages to consider. Smart home devices date back to the 1970s when the X10 protocol was first conceived; this technology allowed for smart home devices to communicate properly [110]. IoT devices in smart homes can be used in a variety of ways, such as measuring home conditions, managing home appliances, and controlling home access [111]. Home automation remains a core feature around which IoT technology is applied [112]. For example, there are numerous home appliances that can be turned on and equipped with IoT technology in order to become more efficient and convenient [113]. There are many benefits that extend beyond convenience. The use of IoT sensors in smart homes can be used to assist the elderly in turning hard-to-reach devices on/off and even detect falls through the use of floor or camera sensors [114]. The market is being driven by the rising popularity of smart devices, such as speakers offered by Amazon and Google. According to a recently released report by Strategy Analytics, the global smart home market has had a positive outcome in recent years [115]. The report further estimates a compound annual growth rate (CAGR) of 10% from 2018 to 2023, leading to a market value of USD 155 billion, as shown in Figure 6.

There are many techniques utilized in order to bring smart home technology to life. One important method relies on radio frequency identification (RFID) systems in order to act as enabler technologies for IoT [116]. RFID is an important technology that helps in identifying objects, recording data, and even controlling individual targets through the use of radio waves [117]. RFID devices can be used in a variety of ways. For example, higher education institutions can utilize RFID technology in student identification cards [118]. RFID technology is also used to detect the indoor roaming activity of elderly individuals and the data collected are used to provide more insight into the health of elderly individuals who live alone [119].

In an ideal future, IoT devices would be able to seamlessly communicate together [7]. There are many challenges that exist in the use of smart home IoT technology. Interoperability is one issue because the cost of using smart home technology is important to consider, and the integration of devices is a concern moving forward [120]. Different technologies utilized by IoT devices in order to create this connectivity include Wi-Fi, ZigBee, Z-Wave, Bluetooth LE, and Thread [121].

Security and privacy are also important to consider because smart grid technology can be a target for cyber attacks [120]. There are so many IoT objects that can be used in homes, and the dynamic and heterogeneous nature of smart home environments presents a challenge when it comes to addressing authentication and privacy issues [52]. Cyber attackers could target items such as smart home routers, gateways, or any other IoT-enabled devices to access data [122]. Many strategies are currently being analyzed in order to address smart home security needs. Blockchain is becoming increasingly utilized because of its benefits of having a decentralized database based on cryptographic techniques [55]). Although blockchain approaches have benefited from decentralized security/privacy, there are drawbacks when it comes to the energy and computational overhead that make it not ideal for IoT devices that are resource-constrained [123].

### 2.4. Smart Cities

Internet of Things devices have many useful applications when it comes to smart cities. A smart city can be understood as a city that is equipped with technology, such as wireless sensor networks and actuators that collect data; it is used to make important decisions in city operations [124]. These systems are inherently complex due to the large number of devices, link layer technologies, and the different services involved in the operation of smart city technology [125]. The smart city concept consists of sensing networks, heterogeneous infrastructure, and information processing systems working together in order to improve a variety of areas within cities [126]. The use of IoT technology to enable smart cities is useful due to the quality of life it provides for the citizens within those cities [127]. The goal of smart cities is to use all of the information that is collected from IoT devices in order to improve the performances of urban services to citizens and also consider resource consumption at the same time [128].

Traffic monitoring is a very important application within the realm of smart cities. It is very common for metropolitan areas to be highly populated, and this causes congestion problems within these cities. Smart cities make use of information communication technologies in order to use the information to make decisions on how to dynamically handle traffic flow [129]. A smart traffic system (STS) involves real-time data collection and requires IoT devices to quickly obtain real-time public traffic data and have it processed [130]. The sensors used in smart traffic management systems can be embedded into roads in order to detect vehicles every 500 or 1000 meters [131]. Cameras are able to apply digital image processing techniques and, consequently, apply algorithms to aid in the prediction of traffic density; this information is then used accordingly [132]. Aside from helping traffic flow, smart traffic management systems also help with improving air quality and providing safety for the elderly [133].

Research indicates that the amount of solid waste will reach around 3.4 billion tons by the year 2050, and that will put a tremendous strain on municipal waste management systems [134]. Smart waste management is another growing application of IoT technology in smart cities. Nowadays, waste management systems are overtaxed and burdened due to the large demands of highly-populated urban areas [135]. The goal of smart waste management is to use IoT devices in order to optimize waste collection and reduce the negative impact on the environment [136]. Major factors driving the need for smart waste management include the demand for energy-efficient processes and the goal of creating healthier environments within cities [137]. A number of different objects can be repurposed into IoT devices, such as trash and recycling containers [138], and different technologies can be used to indicate when it is time to service a full container. In addition to detecting when waste bins are full, some sensors are capable of detecting unpleasant smells using gas sensors [139]. These smart containers essentially work by having sensors in the containers that read, collect, and communicate information about the amount of trash/recycle volume within them in order to better understand when it is time to empty the container [140].

In environmental sector applications, IoT technology has emerged as a valuable tool in enhancing air quality prediction through edge-based computation. Leveraging edge-based computation, IoT devices equipped with sensors collect real-time air quality data at the source, enabling precise prediction models tailored to specific locations. This empowers environmental monitoring systems to deliver accurate and timely information for effective decision-making and pollution control measures.

In the realm of anomaly detection and classification, IoT devices continuously monitor and analyze data from diverse sources, swiftly identify deviations from normal patterns, and recognize potential threats or irregularities. Through advanced machine learning algorithms and localized data processing, IoT systems can rapidly detect anomalies, ensuring the robust security and integrity of IoT networks and the data they generate.

Moreover, multisensory data fusion in multi-application wireless sensor data streams enables the integration of information from various sensors and applications, enabling a comprehensive understanding of intricate IoT environments. By combining data from disparate sources, such as temperature, humidity, and air quality sensors, IoT systems acquire a holistic view of the surroundings, generating valuable insights into informed decision-making across a wide range of applications, spanning from smart cities to industrial automation.

### 2.5. Industry 4.0

**Figure 7:**
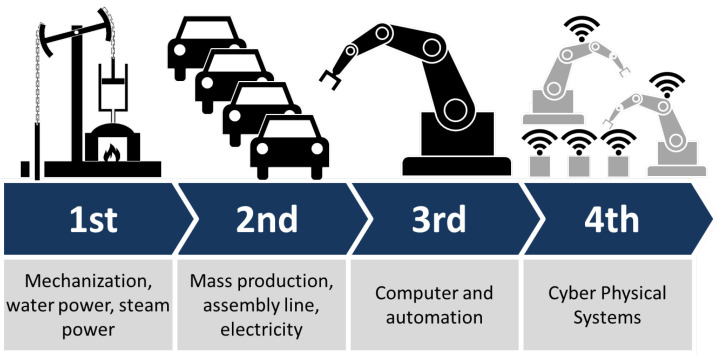
Industry 4.0.

The manufacturing sector is undergoing a revolutionary transformation with the advent of Industry 4.0, ushering in an era of intelligent and interconnected systems. A prominent trend in this domain is the rapid adoption of Industrial Internet of Things (IIoT) devices and sensors [141,142,143,144,145] as shown in Figure 7. These embedded devices empower machines, equipment, and products to gather and transmit real-time data. The data generated by IIoT devices are invaluable for predictive maintenance, enabling manufacturers to proactively identify and address potential equipment failures, thereby reducing downtime and enhancing operational efficiency.

Another significant trend within Industry 4.0 is the heightened focus on cybersecurity. As factories and supply chains become increasingly interconnected and reliant on digital technologies, the need for robust cybersecurity measures has become paramount. Manufacturers are making substantial investments in advanced security solutions to safeguard sensitive industrial data from cyber threats, ensuring the integrity and availability of their systems. This includes implementing encryption techniques, authentication protocols, and intrusion detection systems to fortify critical information.

Artificial intelligence (AI) and machine learning (ML) are also playing crucial roles in driving Industry 4.0 forward. With the copious amounts of data generated by IIoT devices, AI and ML algorithms have the capacity to analyze and derive meaningful insights from vast datasets. Manufacturers are leveraging these technologies to optimize production processes, enhance quality control, and improve decision-making. By detecting patterns and anomalies in real-time data, AI-powered systems can optimize manufacturing operations, identify defects, and propose process improvements, ultimately resulting in increased productivity and reduced costs.

Furthermore, the adoption of cloud computing technologies has empowered manufacturers to securely store and access vast quantities of data in a flexible and scalable manner. Cloud-based platforms provide the agility required for data analysis, collaboration, and remote monitoring. Manufacturers can conveniently access real-time production information from anywhere, enabling remote troubleshooting, predictive maintenance, and streamlined supply chain management.

Collaborative robots, also known as cobots, represent another noteworthy trend in Industry 4.0. These robots work alongside human operators, assisting them in various tasks and augmenting productivity. Cobots, which are designed to be safe, adaptable, and easily programmable, can adapt to changing production requirements. They are adept at handling repetitive and physically demanding tasks, freeing up human workers to concentrate on more intricate and creative aspects of the manufacturing process.

The integration of virtual reality (VR) and augmented reality (AR) technologies is also gaining momentum within Industry 4.0. These immersive technologies offer interactive and intuitive interfaces for training, simulation, and maintenance purposes. VR and AR enable workers to visualize and manipulate virtual representations of machinery, products, and processes, thereby enhancing training effectiveness and reducing errors.

Industry 4.0 is driving a transformative shift in the manufacturing landscape. The widespread adoption of IIoT devices, AI and ML algorithms, cloud computing, cybersecurity measures, cobots, and immersive technologies is reshaping traditional industrial processes into highly connected, intelligent, and efficient operations. Manufacturers who embrace these trends are poised to reap numerous benefits, including improved productivity, cost reductions, enhanced product quality, and increased agility in an intensely competitive global marketplace.

## 3. Challenges and Active Research Topics

### 3.1. Security of IoT Devices

**Figure 8:**
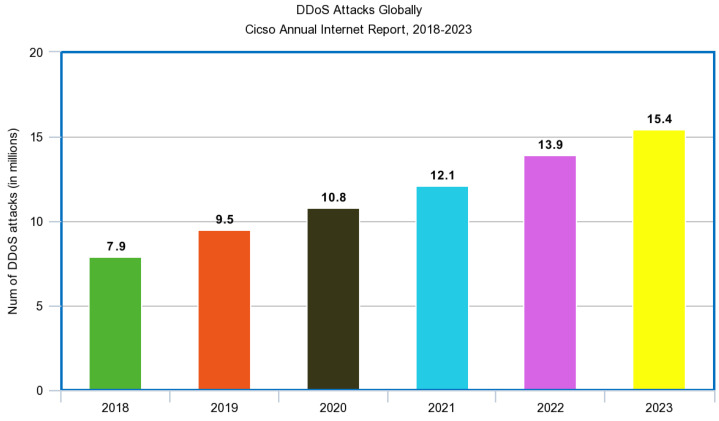
DDoS attacks worldwide.

The use of Internet of Things devices is progressively becoming more prevalent in the daily lives of people around the world; however, cyberattacks remain a large threat to the safe use of IoT [146]. Different examples of these devices are mobile phones, alarms, medical sensors, smartwatches, security systems, and more. The use of these devices continues to expand, and the need for strong security is vital to their success. Although these devices bring convenience, they come with many security issues and vulnerabilities. Since IoT devices often collect sensitive data, these data transmissions can be intercepted by third parties who intend to conduct harm or use these data for nefarious purposes. In one case, there were even attacks that could target IoT devices, such as the Mirai malware [147], which would hack/convert devices into its botnet and carry out DDoS attacks [148] as shown in Figure 8. Malware, such as the Mirai and others, take advantage of the vast amounts of poorly protected IoT devices, which commonly suffer from poor configurations and open designs, making them targets [149]. Detecting malware and IoT botnets remains an active research area, and many techniques are being applied. The use of a lightweight approach in the classification of IoT malware through the use of image recognition is just one example [150]. Other ways include the use of machine learning algorithms that use supervised, unsupervised, and reinforcement learning in order to handle tasks such as authentication, access control, and malware detection [151].

There is an enormous amount of data being transmitted on a daily basis, which impacts critical operations across many applications. It could be possible for attackers to disrupt entire networks that rely on IoT technology, and the consequences would be devastating [152]. Hackers and criminals could seriously impact the expansion of IoT devices in the future, and it is crucial that security be thoroughly researched to mitigate these negative impacts.

### 3.2. Authentication and Password Security

One security issue is the lack of security in regard to authentication and passwords. Many IoT devices rely on password security in order to stay protected from cyber criminals who are attempting to gain access to them. These passwords can often be weak, and criminals can have easy access to IoT devices. There is a lack of standardization revolving around how complex passwords should be. Research shows that having more complex password combinations in IoT devices can prevent more cyber attacks [153]. Even with stronger passwords, there would need to be additional security measures to prevent cyber attackers.

One example of an additional layer of security involves the use of multi-authentication in IoT devices. In order to authorize the correct users to access IoT devices, it is a prerequisite to first have authentication [154]. Authentication is the process by which the identity of users is verified before allowing them to gain access to a system. Passwords represent just one level of authentication, and it is important to understand the other types of authentication mechanisms that IoT devices can take advantage of. Different methods, such as the utilization of elliptic curve cryptography, can be useful when performing authentications in IoT security systems [155].

One-time passwords can sometimes be useful for authentication purposes related to IoT devices. These passwords work by having a private key generator (PKG) generate a one-time password, and this password is used as a private key that is needed in order to gain access. The last phase of using one-time passwords involves validation. In this phase, the application and IoT device exchange data via a one-time password, verifying that the password was sourced from a valid location [56].

### 3.3. Interoperability Challenges

Using IoT devices smoothly and without compatibility issues remains a challenge both now and in the future. Interoperability is important because it allows IoT devices to communicate with each other more efficiently. It is a challenge to have IoT devices work together seamlessly because many operate on different infrastructures, devices, APIs, and even data formats [156]. The need for the safe interoperability of IoT devices has even led to the creation of international organizations that develop standards that IoT devices can adopt with the intention of becoming more compatible [157]. The use of protocols and standards, such as Bluetooth and ZigBee, is critical to the rise of IoT technology because it essentially establishes the rules for use and communication, helping to address interoperability concerns [158]. International organizations, such as IEEE, the Internet Engineering Task Force (IETF), OneM2M, and others, have developed important standards and protocols and play integral roles in influencing the IoT [159]. One important contribution to addressing these challenges is the BiG IoT project, which is an initiative that seeks to create a common API that different IoT devices could communicate through [160].

### 3.4. Cloud Computing Research

Since IoT devices generate massive amounts of data, cloud computing solutions are continually being used and researched in order to better handle these data demands. Cloud IoT is a novel IT paradigm that represents the merging of the cloud with IoT, and research in this area will be crucial in moving this technology forward [161]. Cloud computing is essentially an extension of distributed computing, parallel computing, and grid computing [126]. There are many IoT constraints that cloud computing addresses, such as processing, storage, and communication [162]. For example, a major concern about IoT data involves the security risks that come from storing data locally on IoT devices [163], and cloud computing aims to address this concern by allowing data storage in cloud computing servers [164]. Since data collected from IoT devices are often unstructured, cloud computing research looks to improve the real-time data processing capabilities and allow for more dynamic resource management [165].

### 3.5. Potential Future Directions and Developments

The potential future directions and developments of IoT in healthcare, agriculture, smart homes, smart cities, and Industry 4.0 are poised to bring about transformative changes and advancements. In healthcare, the integration of IoT with telemedicine, wearable health technology, and advanced data analytics holds promise for revolutionizing patient care. Real-time monitoring, personalized treatment plans, and early disease detection can be facilitated through IoT-enabled devices, leading to improved health outcomes. In agriculture, IoT-driven precision farming techniques, such as smart irrigation and crop disease detection, have the potential to optimize resource utilization, conserve water, and enhance crop yield. By leveraging real-time data from IoT sensors, farmers can make informed decisions and implement timely interventions. In the realm of smart homes, the focus will be on seamless integration, energy efficiency, and personalized automation. IoT-enabled smart home solutions will allow for centralized control and management of various devices, optimizing energy consumption and providing tailored experiences for inhabitants. In smart cities, IoT applications will enhance transportation systems, environmental monitoring, and citizen engagement. Intelligent traffic management, real-time tracking of air quality, and participatory governance will contribute to improved mobility, sustainability, and quality of life. Finally, Industry 4.0 will witness the integration of IoT in industrial automation, predictive maintenance, and supply chain optimization. IoT-driven technologies will enable the real-time monitoring of machines, predictive maintenance strategies, and streamlined logistics, leading to enhanced productivity and reduced downtime. Continued research and development in these areas will shape the future of IoT, paving the way for innovative solutions and transformative advancements across sectors.

## 4. Conclusions

IoT technology has rapidly emerged as a revolutionary field in computer science, facilitating the connection of everyday objects to the internet and enabling a vast network of interconnected devices. The widespread adoption of IoT devices has been fueled by advancements in wireless networking technologies and the increasing demand for automation and efficiency across multiple industries. This technology is being utilized across various sectors, and its utilization is expected to continue expanding. IoT is progressively integrating into society and has become particularly significant in areas such as healthcare, agriculture, smart homes, smart cities, and more. The progress in technological and networking capabilities underscores the pivotal role of emerging technologies in driving the proliferation of IoT worldwide. However, despite its potential, there are several challenges, including security and privacy concerns, which researchers must address through innovative approaches. As networking and technological advancements continue to support the rise of IoT, its applications will further grow and become increasingly integrated into our societal fabric. This paper has examined the emergence of IoT devices, explored their common applications in healthcare, agriculture, and smart cities, and delved into the future prospects of this promising field.

The future of IoT technology holds tremendous promise. Advancements in machine learning and artificial intelligence will play vital roles in driving innovation in this field, unlocking new possibilities for IoT applications. However, it is essential to tackle security challenges, enhance affordability, and raise awareness and knowledge among users and stakeholders to ensure the sustained growth and success of IoT technology.

To summarize, IoT technology has brought about a revolution in various industries, offering vast opportunities for automation, efficiency, and improved decision-making. By comprehending the driving forces behind the emergence of IoT devices and exploring their applications in different fields, researchers and practitioners can shape the future of IoT and harness its potential for the betterment of society.

## Data Availability

Not applicable.

## Abbreviations

The following abbreviations are used in this manuscript:

IoT	Internet of Things
IoMT	Internet of Medical Things
DDOS	distributed denial of service
RFID	radio frequency identification
STS	smart traffic systems
PKG	private key generator
IETF	Internet Engineering Task Force
